# Relaxin Level in Patients With Atrial Fibrillation and Association with Heart Failure Occurrence: A STROBE Compliant Article

**DOI:** 10.1097/MD.0000000000003664

**Published:** 2016-05-27

**Authors:** Hao Zhou, Xiang Qu, Zhan Gao, Gaoshu Zheng, Jie Lin, Lan Su, Zhouqing Huang, Haiying Li, Weijian Huang

**Affiliations:** From the Department of Cardiovascular Medicine, the First Affiliated Hospital of Wenzhou Medical University; The Key Lab of Cardiovascular Disease of Wenzhou, Wenzhou, China.

## Abstract

Atrial fibrillation (AF) is the most common arrhythmia requiring medical treatment and has been associated with enhanced atrial fibrosis and heart failure (HF). Relaxin (RLX), an antifibrosis and antiinflammatory peptide hormone, may be used to evaluate atrial fibrosis and is associated with HF occurrence in AF. We aimed to clarify the clinical significance of RLX level in patients with AF.

We measured circulating levels of RLX and other fibrosis-related factors in 311 patients with sinus rhythm (SR; n = 116) or AF (n = 195). All discharged AF patients were followed up for the occurrence of HF for a mean of 6 months.

Circulating levels of RLX were significantly different in patients with AF as compared with SR (*P* < 0.001), and in the subgroup analysis of AF. RLX level was correlated with left atrial diameter (LAD; *R* = 0.358, *P* < 0.001). Among followed up AF patients, on Kaplan–Meier curve analysis, patients with the third RLX tertile (T3) had a significantly higher HF rate than those with the 1st tertile (T1) (*P* = 0.002) and the cut-off value was 294.8 ng/L (area under the ROC curve [AUC] = 0.723). On multivariable analysis, HF occurrence with AF was associated with increased tertile of serum RLX level (odds ratio [OR] 2.659; confidence interval [95% CI] 1.434–4.930; *P* = 0.002).

RLX is associated with fibrosis-related biomarkers and significantly elevated in AF. RLX was related to the HF occurrence in patients with AF.

## INTRODUCTION

Atrial fibrillation (AF), associated with mortality, morbidity, and high health care costs, affects millions of people worldwide and is increasing in prevalence.^1,2^ Abnormalities in atrial structure, or remodeling, play an important role in the development of AF. Among the most consistently described of these structural abnormalities is atrial fibrosis.^3^

Atrial fibrosis represents collagen and extracellular matrix deposition within the atria, often resulting in heterogenous conduction and impaired contraction.^4^ Atrial fibrosis is associated with AF in both animal models^5^ and humans.^6–8^ Most studies have involved in vitro tissue or explanted tissue, with little translation to the clinic. Delayed enhancement magnetic resonance imaging as a semiquantitative method of quantification has shown promise, but quantifying and relieving atrial fibrosis in AF remains limited.^9^

AF and congestive heart failure (HF) are commonly encountered together, and either condition predisposes the patient to the other condition. Congestive HF and AF share common mechanisms, including myocardial fibrosis and inflammatory response.^10^ Several risk factors common to both include age, hypertension, valve disease, and myocardial infarction as well as various medical conditions and genetic variants,^11^ but we lack an exact predictor of HF in AF.

Relaxin (RLX), as a naturally occurring human hormone, can downregulate the deposition of collagen and other extracellular matrix proteins. A number of downstream pathological processes are involved, including reduced expression of transforming growth factor-β (TGF-β) and tumor necrosis factor-α (TNF-α), and increased activity of matrix metalloproteinases (MMP),^12^ which results in reduced fibrosis. Some animal experiments have demonstrated that RLX could inhibit both the proliferation of cardiac fibroblasts and the synthesis of collagen.^13^ Furthermore, RLX has been investigated as treatment for HF in clinical trials and found to have good safety and tolerability in recent investigations.^14^

We speculated that RLX might have a key role in reflecting atrial fibrosis in AF and hypothesized that RLX level is related to HF occurrence in AF. Here, we measured levels of RLX and related indicators in patients with sinus rhythm (SR) and AF to understand the changes in levels of these proteins in AF and the clinical significance of RLX in patients with AF.

## METHODS

### Study Design and Patients

We included totally 311 consecutive patients with SR (n = 116) or AF (n = 195) from the first affiliated hospital of Wenzhou Medical University. Patients were assigned to the “SR group” provided that their basic rhythm was SR without antiarrhythmic therapy and there was no evidence for AF in previous medical documentation or in electrocardiography, dynamic electrocardiography, or cardiac telemetry system results during hospitalization. Patients were assigned to the “AF group” based on previous medical documentation and electrocardiography, dynamic electrocardiography, or cardiac telemetry system results performed during hospitalization. Converting AF to SR in 7 days indicated a diagnosis of paroxysmal AF (PaAF); otherwise, duration >7 days indicated a diagnosis of persistent AF (PeAF). Key exclusion criteria were severe HF needing dialysis or rescue breathing machine, hemodynamic instability and myocardial infarction of Killip 3–4 needing a breathing machine or intraaortic balloon pump support, because these acute exacerbation states may influence the levels of biomarkers. All discharged AF patients were followed up for 5 to 7 months to identify the occurrence of HF. Each AF and HF episode were blindly adjudicated by a validation committee referring to Guidelines for the Management of Atrial Fibrillation: the Task Force for the Management of Atrial Fibrillation of the European Society of Cardiology (ESC) and ESC Guidelines for the Diagnosis and Treatment of Acute and Chronic Heart Failure 2012.

At baseline, patients completed a standardized questionnaire to assess risk factors, including age, sex, duration of AF, smoking, alcohol intake, hypertension, diabetes, and myocardial infarction. Physical examination included blood pressure, heart rate, body mass index, and resting electrocardiography. Laboratory measurements included levels of serum creatinine, transaminase, low-density lipoprotein cholesterol, fasting blood glucose, C-reactive protein, and brain natriuretic peptide (BNP), which was measured by a chemiluminescence method, and D-dimer measured by the immunoturbidimetric method. Ultrasonic cardiography measurements included left ventricular ejection fraction, left atrial diameter (LAD), left ventricular end-diastolic diameter, pulmonary arterial pressure, and cardiac output. In our study, we used the M mode to measure the anteroposterior diameter of the left atrium as LAD in the parasternal long-axis view perpendicular to the aortic root long axis by independent team of echocardiography according to the Recommendations for Cardiac Chamber Quantification by Echocardiography in Adults: An Update from the American Society of Echocardiography and the European Association of Cardiovascular Imaging.^15^ Fibrotic, antifibrotic, and inflammatory markers measured included RLX, TGF-β, TNF-α, MMP-2, and procollagen type I C-terminal peptide (PICP), released from the synthetic process of collagen I.

This study was reviewed and approved by the Ethics Review Board of the First Affiliated Hospital of Wenzhou Medical University. We obtained written informed consent from all the participants in this study.

### Biomarker Measurements

Blood was taken from the peripheral vein on the 2nd day morning of hospitalization with patients in a stable state. Serum was extracted and stored at −80 °C. Samples were analyzed when 90 samples were accumulated or storage time reached 1 month. RLX, MMP-2, TGF-β, TNF-α, and PICP levels were measured by enzyme-linked immunosorbent assay kits (Boyun, Shanghai, China). The sensitivity (lower detection limit) for assays was for RLX, 10 ng/L; MMP-2, 2 ng/mL; TGF-β, 10 ng/L; TNF-α, 3ng/L; and PICP, 2 ng/mL. All samples were run in duplicate and measured at 450λ. Other biochemical measurements were performed at the laboratory of the hospital.

### Statistical Analysis

SPSS20.0 (SPSS Inc., Chicago, IL) was used for statistical analysis. Continuous variables are presented as mean ± SD, and categorical variables as frequencies and percentages. Continuous variables were compared by 1-way ANOVA; the Student–Newman–Keuls test was used for analyzing intergroup differences and the Chi-square test for categorical variables. Correlations between 2 parameters were assessed by simple linear regression. Receiver operating characteristic (ROC) curves were used to assess the clinical application for RLX. The proportion of patients free of HF in AF was plotted by the Kaplan–Meier method, with statistical significance examined by the log-rank test. Predictors of AF occurrence were examined by univariate and multivariable logistic regression analysis. Two-sided *P* < 0.05 was considered statistically significant.

## RESULTS

### Patient Characteristics

A total of 311 patients (age 39–91 years) were studied. The included patients had SR (n = 116), PaAF (n = 80), and PeAF (n = 115). The baseline subject characteristics are in Table 1. At baseline, the mean heart rate was greater for PeAF than SR patients (*P* < 0.001) and duration of AF was longer for PeAF than PaAF patients (*P* = 0.006). LAD was greater for PeAF than other patients (*P* < 0.001). There were no significant differences among 3 groups in other characteristics.

**TABLE 1:**
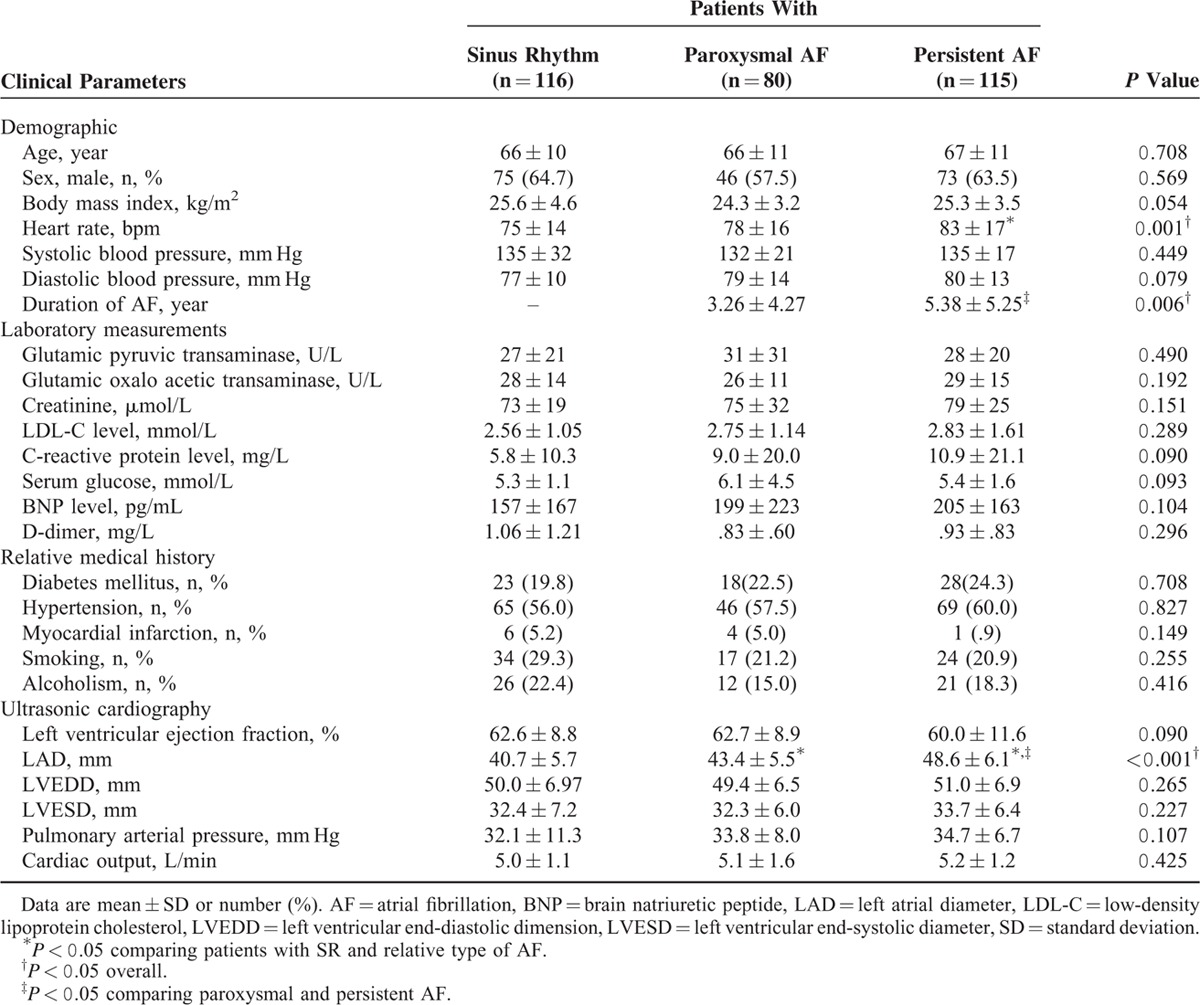
Clinical Characteristics of Patients With Atrial Fibrillation

### Serum Concentration of Fibrosis-Related Markers

**FIGURE 1:**
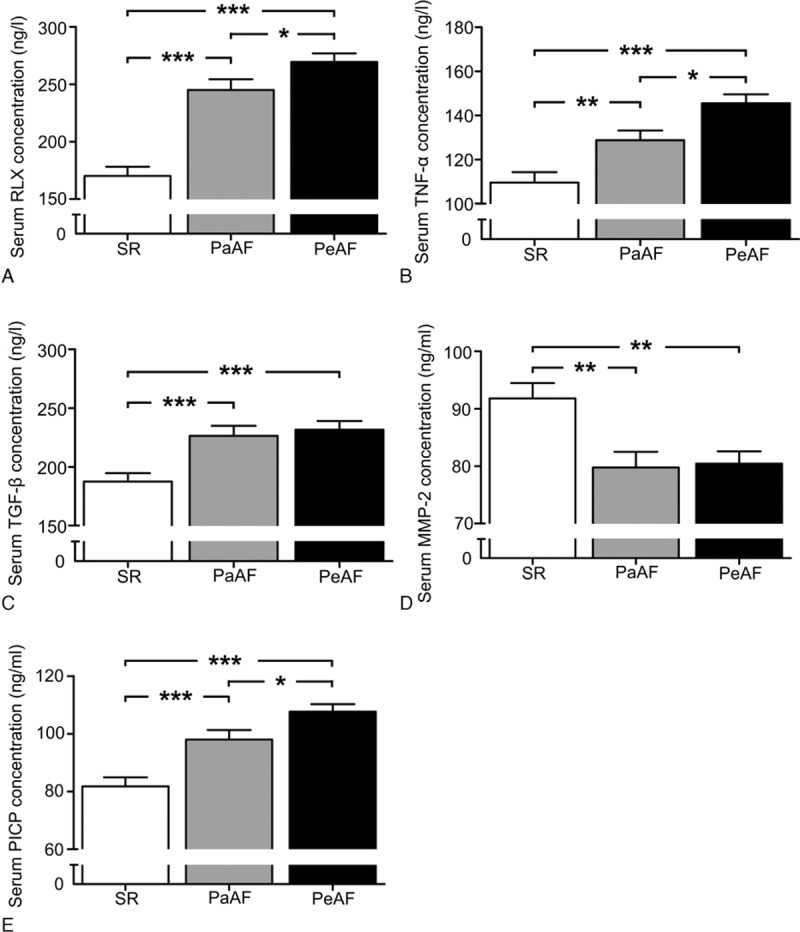
Serum concentrations of (A) RLX, (B) TNF-α, (C) TGF-β, (D) MMP-2, and (E) PICP in patients with SR, PaAF, and PeAF. Data are mean ± SEM. ^∗^*P* < 0.05; ^∗∗^*P* < 0.01; ^∗∗∗^*P* < 0.001. MMP-2 = matrix metalloproteinase-2, PaAF = paroxysmal atrial fibrillation, PeAF = persistent atrial fibrillation, PICP = procollagen type I C-terminal peptide, RLX = relaxin, SEM = standard error of the mean, SR sinus rhythm, TGF-β = transforming growth factor-β, TNF-α = tumor necrosis factor-α

Levels of fibrosis-related biomarkers (RLX, TNF-α, TGF-β, MMP-2, and PICP) significantly differed among the 3 groups (*P* < 0.001) (Table 2). We 1st determined circulating RLX levels as a marker reflecting the extent of fibrosis of AF. As compared with SR patients, as shown in Figure 1, PeAF patients showed markedly increased levels of RLX (*P* < 0.001), TNF-α (*P* < 0.001), TGF-β (*P* < 0.001), PICP (*P* < 0.001), and reduced MMP-2 activity (*P* = 0.001). Level of RLX (*P* < 0.001), TNF-α (*P* = 0.004), TGF-β (*P* = 0.001), and PICP (*P* = 0.047) was higher and MMP-2 (*P* = 0.001) was lower for PaAF than SR patients. The level of RLX, TNF-α, and PICP in PaAF patients was in the middle of that for SR and PeAF patients.

**TABLE 2:**
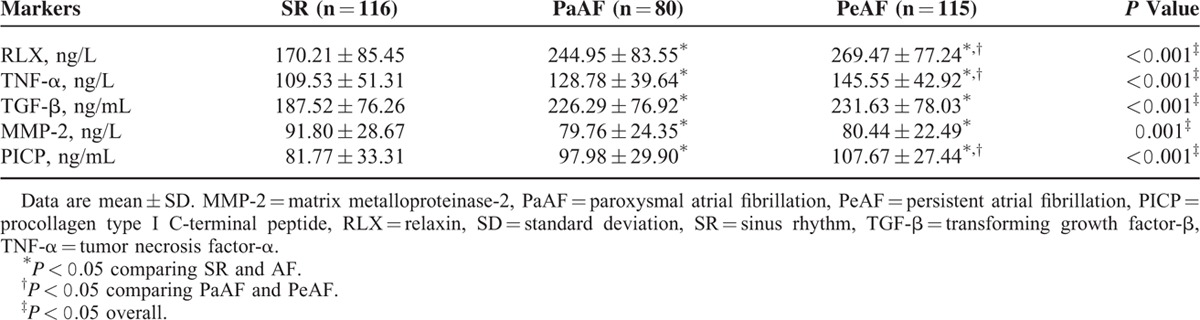
Serum Levels of Fibrosis-Related Markers for Patients With Sinus Rhythm and Atrial Fibrillation

### Correlation of Levels of Fibrosis-related Markers

**FIGURE 2:**
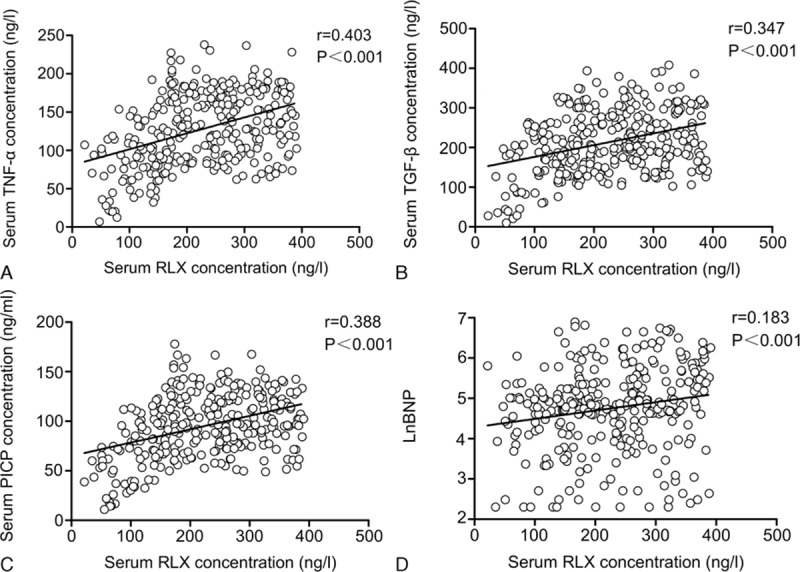
(A) Correlation between mean serum RLX and (A) TNF-α, (B) TGF-β, (C) PICP, and (D) lnBNP. lnBNP = log base-e transformation for BNP, PICP = procollagen type I C-terminal peptide, RLX = relaxin, TGF-β = transforming growth factor-β, TNF-α = tumor necrosis factor-α.

We investigated a connection between circulating levels of RLX and other fibrosis-related biomarkers in Figure 2. We found serum concentrations of RLX with a significant positive correlation between TNF-α (*R* = 0.403, *P* < 0.001), TGF-β (*R* = 0.347, *P* < 0.001), and PICP (*R* = 0.388, *P* < 0.001). Serum RLX level was not correlated with MMP-2 level (*R* = 0.079, *P* = 0.165). We assessed whether RLX was correlated with the level of BNP, with log base-e transformation for BNP (lnBNP) because it did not exhibit a normal distribution. lnBNP level was correlated with levels of RLX (*R* = 0.183, *P* < 0.001).

### Elevated RLX Expression With Left Atrial Dilatation

**FIGURE 3:**
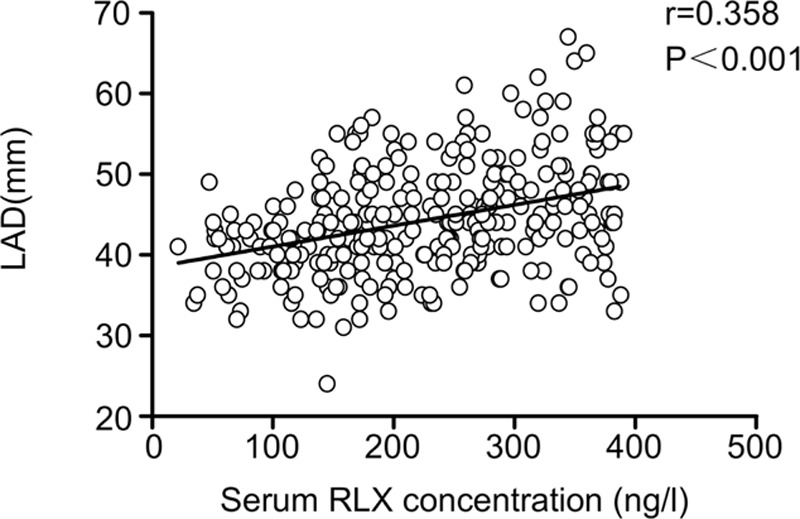
Correlation between mean serum RLX concentration and LAD. LAD = left atrial diameter, RLX = relaxin.

We examined whether left atrial dilatation was associated with upregulation of RLX. We established a significant positive linear correlation between RLX level and elevated LAD (*R* = 0.358, *P* < 0.001) in Figure 3. We found no significant correlation between serum concentrations of RLX and left ventricular end-diastolic dimension (*R* = 0.083, *P* = 0.159).

### High RLX Level Was Correlated to HF Occurrence in AF

**FIGURE 4:**
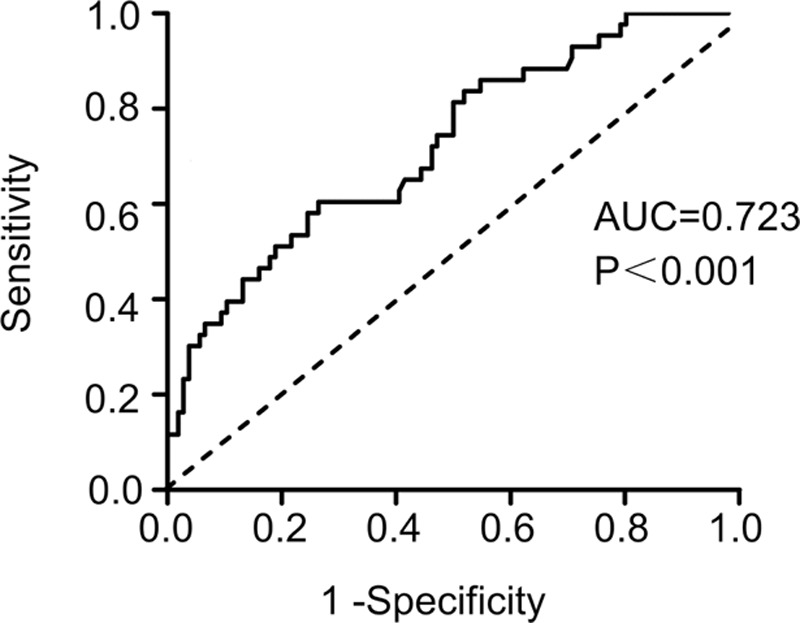
ROC curve of relaxin for assess the clinical application of predicting heart failure (AUC = 0.723). AUC = area under the ROC curve, ROC = receiver operating characteristic

**FIGURE 5:**
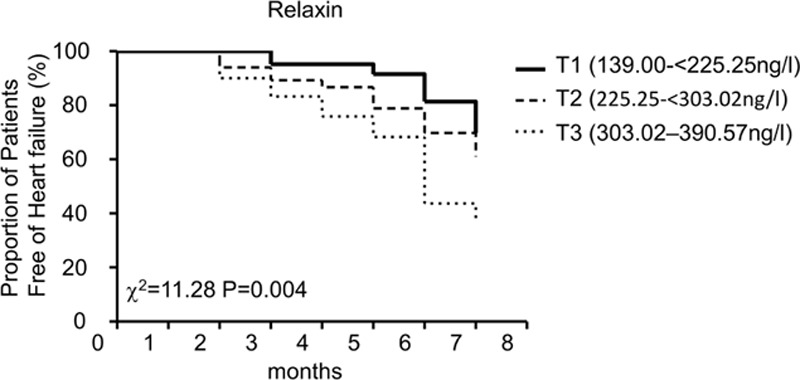
Kaplan–Meier curve analysis. Proportion of patients free of heart failure in atrial fibrillation according to tertile of serum relaxin level. Heart failure rate was statistically significant among 3 groups (log-rank test, χ^2^ = 11.28, *P* = 0.004). Post hoc analysis showed a significant difference in heart failure rate between 1st tertile (T1) and 3rd tertile (T3) patients (χ^2^ = 9.788, *P* = 0.002).

Next, we examined whether serum RLX can be used to early predict HF occurrence in AF. For mean follow-up period was 6 months (range 5–7 months). Forty-three cases occurred HF in AF patients. We divided AF patients into 3 groups according to the trisection of AF RLX concentration: 1st tertile [T1; 139.00–<225.25 ng/L], 2nd tertile [T2; 225.25–<303.02 ng/L], and 3rd tertile [T3; 303.02–390.57 ng/L]. In T1, T2, and T3, the HF occurred respectively 6 cases, 13 cases, and 24 cases. The mean RLX level was 177.42 ± 22.92, 266.59 ± 20.53, and 348.37 ± 24.31 ng/mL, respectively. Figure 4 shows the ROC curves of RLX for assessing the clinical application of predicting HF. When the circulation RLX concentration cut-off value was 294.8 ng/L, the sensitivity was 60.47% (95% CI, 44.41%–75.02%) and the specificity was 73.58% (95% CI, 64.13%–81.68%). The area under the ROC curve was 0.723 (95%CI, 0.633–0.812). As shown in Figure 5, HF rate significantly differed among the 3 groups according to Kaplan–Meier curve (log-rank test, χ^2^ = 11.28, *P* = 0.004). On post hoc analysis, the difference in HF rate between T1 and T3 patients was significant (χ^2^ = 9.788, *P* = 0.002).

On univariate analysis, the occurrence of HF was associated with increased LAD (OR, 1.125; 95% CI, 1.055–1.199; *P* < 0.001), PeAF (OR, 2.698; 95% CI, 1.232–5.907; *P* = 0.013), and increased tertile of serum RLX level (OR, 2.581; 95% CI, 1.576–4.228; *P* < 0.001) (Table 3). On multivariable analyses, the occurrence of HF was associated with increased LAD (OR, 1.159; 95% CI, 1.047–1.283; *P* = 0.004) and increased tertile of serum RLX level (OR, 2.659; 95% CI, 1.434–4.930; *P* = 0.002).

**TABLE 3:**
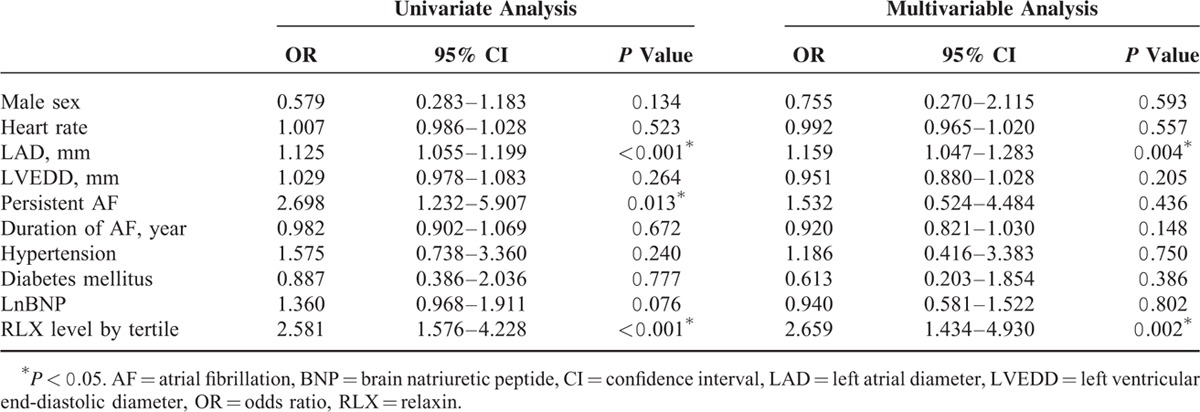
Univariate and Multivariable Analyses of Predictors of Heart Failure Occurrence

## DISCUSSION

### AF and Atrial Fibrosis

AF is the most common arrhythmia and recently has been found associated with an increased risk of silent cerebral infarct, stroke, and autonomic dysfunction in type 2 diabetes.^16,17^ Atrial fibrosis is a common pathological condition seen in AF, especially peAF. The link between atrial fibrosis and AF was demonstrated in several animal models^18–20^ and human studies. In humans, increased collagen deposition was documented in patients with lone-AF as compared with normal SR,^21^ and atrial fibrosis was also observed in AF with potential structural disease such as mitral valve disease and cardiomyopathy as compared with SR patients.^22^ In terms of epigenetics, histone deacetylases have important functions in regulating cardiac gene expression and are associated with AF and fibrosis. Histone deacetylase inhibition reversed atrial fibrosis and arrhythmic inducibility in HopX transgenic mice with left ventricular hypertrophy.^23^ Recent research had found that the molecular mechanisms related to fibrotic alterations may be necessary to understand the AF substrate alterations before to choose the best therapeutical management. Sardu et al^24^ had focused on microRNAs plasma levels in AF patients treated by catheter ablations and had found the microRNAs modulated by catheter ablation (downregulation and/or upregulation) were related to AF structural processes. Therefore, they considered microRNAs, as an AF biomarkers, may differentiate responders versus nonresponders to an ablative approach. All these studies highlight the association between atrial fibrosis and AF. Thus, attenuating atrial fibrosis may be a plausible approach to treating AF.^3^ However, detection of atrial fibrosis remains a challenge. In our study, we used PICP, a peptide released with synthetic process of collagen I, which constitutes more than 80% of the myocardial interstitium,^25^ as a biomarker of atrial fibrosis. In this study the serum PICP level increased, which supported the mechanism of AF. Previous research also supports increased serum PICP levels positively associated with the development of AF.^7^

### Fibrotic and Antifibrotic Biomarkers and Clinical Relevance

RLX as an endogenously reproductive hormone that has extracellular matrix remodeling effects, potent antifibrotic activity, and other cardio-protective actions.^26^ In rat models of hypertension and diabetic cardiomyopathy, RLX significantly inhibited TGF-β-mediated collagen production and cardiac fibrosis.^27,28^ Meanwhile, RLX has been found to promote MMP expression and activity, while inhibiting the actions of tissue inhibitors of MMP to induce collagen breakdown.^29^ It also decreased the release of TNF-α in response to an adjuvant and endotoxin.^30,31^ In this study, we found that in patients with PeAF, the levels of RLX, TNF-α, TGF-β, and PICP were upregulated and the level of MMP-2 was downregulated, and TNF-α, TGF-β, and PICP levels were positive correlative with RLX. These shown that the RLX was closely related with fibrosis. In chronic HF patients, a research confirmed that plasma H2RLX increased even significant correlation with collagen.^32^ Although the own secretion of RLX might insufficient to modify extracellular matrix deposition and turnover the synthesis and deposition of collagen. Therefore, exogenous application of RLX may relieve fibrosis. In the present study, recombinant human RLX was used to inhibit both the proliferation of cardiac fibroblasts and the synthesis of collagen under the high glucose condition in vitro.^33^ Moreover, RELAX-AHF study had discovered serelaxin was associated with improvement in several acute HF clinical outcomes and proved its security in acute HF.^34,35^

### Elevated RLX Expression and Atrial Dilatation

Whether the elevated level of RLX in patients with AF was associated with dilatation of atria remains unknown. This study revealed that RLX level was proportional to LAD and lnBNP in AF, which similar to the research of Dschietzig et al in congestive HF.^36^ Various causes result in atrial dilatation finally might raise the secretion of RLX, meanwhile upregulated the serum of BNP. Although atrial remodeling was the most common phenomenon of atrial fibrillation. RLX level was elevated in larger LAD, which may suggest that RLX was associated with the progression of LAD in AF.

### Upregulation of RLX Level to Predict HF Occurrence in AF

From recent findings, AF and HF are interdependently relationship. Patients with one of the disorder will increase the risk of another illness. When the AF and HF occur together rather than either alone will carry an observably worse prognosis.^10^ In addition, the abnormal hemodynamics of AF, including long-term elevated heart rate, increased atrial filling pressure, invalid atrial contraction, and the lack of synchrony in atrioventricular contraction, will impair ventricular function and contribute to symptoms of congestive HF.^10,37^ Nevertheless, our ability to early predict HF occurrence in AF remains limited. However, we found an association of increased RLX level and increased rate of occurrence HF in AF. LAD was also an independent risk factor of HF occurrence in AF.

There are some limitations to this study. First, we did not distinguish atrial fibrosis and ventricular fibrosis, so the PICP results should be interpreted with caution. Second, blood specimens were collected only once during hospitalization, so we unable to provide the daily variability and changes in RLX levels after discharge. Still some other confounders of RLX cannot be excluded. Finally, in this study, we discovered the mean heart rate was greater for PeAF than SR patients. However, we need more data to realize the relationship between heart rate variability and HF recurrence. Hence, in our further study, we will observe heart rate variability. Further studies will be needed to fully characterize the panoply of the actions of RLX

In conclusion, RLX is associated with fibrosis-related biomarkers. Serum RLX level differed among 3 groups of patients (SR, PaAF, and PeAF) and arose with the development of AF. PICP and LAD were positively associated with RLX level. High RLX level, as well as LAD, was correlated with occurrence of HF in AF. Although research into the fundamentals of structural remodeling has not been exhausted, the use of biomarkers involved in atrial fibrosis may be a promising new approach.
